# Exploring psychosis and bipolar disorder in women: a critical review of the qualitative literature

**DOI:** 10.1186/s12888-014-0281-0

**Published:** 2014-11-18

**Authors:** Anja Wittkowski, Laura K McGrath, Sarah Peters

**Affiliations:** University of Manchester, School of Psychological Sciences, Zochonis Building, Brunswick Street, Manchester, UK; Manchester Mental Health and Social Care Trust, Manchester, UK

**Keywords:** Metasynthesis, Psychosis, Women, Mental health, Illness

## Abstract

**Background:**

The experiences of women with severe mental illness warrant particular consideration to identify the strategies they use to facilitate recovery. This review systematically examined women’s experiences of psychosis and bipolar disorder.

**Methods:**

Following an extensive database search, 13 studies met inclusion criteria. Noblit and Hare’s metasynthesis approach was used to synthesise these qualitative studies exploring the experiences of 250 women, of which 78 (31.2%) were also mothers.

**Results:**

Twelve sub-ordinate themes were identified and categorised into three overarching themes: 1) women’s beliefs about illness, 2) perceived consequences of illness, and 3) strategies used to cope with illness. Contextual factors and spiritual beliefs were found to be important in these women’s illness appraisals. Women incorporated diagnosis-related information into illness models if it was concordant with their existing beliefs.

**Conclusions:**

Women reported negative illness consequences relating to stigma, loss of self-determination and changes to relationships. They employed various strategies in order to cope with illness. Barriers to strategy use and clinical recommendations are presented.

## Background

Extensive research has been undertaken exploring the experiences of people with mental illness [1]. Psychosis and bipolar disorder have been assigned to the general category of ‘serious mental illness’ (SMI). Bipolar disorder is characterised by a primary disruption in mood. It is one of the leading causes of disability worldwide, affecting 1–1.5% of the population in the United States (US) and the United Kingdom (UK) [2]. With alternating phases of mania and depression, bipolar disorder can have a serious impact on functioning [3].

The most common diagnosis associated with psychosis is schizophrenia, which affects 1% of the population in all cultures. Symptoms include hallucinations and/or delusions, social withdrawal, flat affect and a loss of sense of pleasure and motivation [4]. Almost equal numbers of men and women are affected by psychosis, but onset is later for women [5-7].

Initially, psychosis and bipolar disorder were assumed to be distinct clinical entities [8] but in recent guidelines [9] bipolar disorder is referred to as a psychotic disorder.^Note: the term psychosis will be used throughout.^

Evidence suggests that gender differences exist in the experience of mental illness. For example, women use mental health services more frequently than men and they desire a wider range of treatment options [10]. However, the diversity within women with mental illness has been overlooked [11]. Women’s disadvantage within the mental health system interacts with other positions of disadvantage [12]. For example, exposure to risk factors for psychosis, such as abuse, is more likely to be found in women who are socially marginalized, such as those from lower economic status (SES) or black and minority ethnic groups (BME) [13].

While the impact of marginalization on risk factors has been well documented, there is evidence that mental health services respond to different groups of women in different ways, both in terms of accessibility of services and treatments received [14]. Societal attitudes towards women with mental illness compound this disadvantage. Stigma against people with mental illness has been named as one of the most important challenges for those experiencing such difficulties [15]. Goffman [16] identified two levels of stigma: the discredited and the discreditable. An individual with a mental illness would be viewed as discreditable because their undesirable attribute is not apparent to others initially and they can engage in behaviours designed to manage identity. In discredited individuals, stigma is apparent to others and cannot be concealed.

Finally, women’s roles as wife, mother and carer of elderly relatives, as well as paid employee, need to be taken into account to understand the risk factors for mental illness and the consequences for women and their families [17]. Given the impact that psychosis can have on functioning, there is concern for women with a parenting role. It is estimated that internationally around 60% of women with SMI have dependent children and that the majority parent their children adequately [18]. However, some described significant difficulties [19], without adequate support from health providers [20,21].

When parenting is compromised, children’s development might be affected. Although some children might not experience any difficulties [22], children with a parent with SMI are at greater risk for developing a range of problems including relationship difficulties, mental illness, developmental delay and lower academic attainment [23]. Some women with SMI might be unable to maintain custody [24].

### Aims of the study

As it is important to consider the views of service users in developing policy and services [25,26], the aim of this metasynthesis was to review qualitative research exploring women’s experiences of psychosis and to extend the interpretative possibilities offered by the current literature, enabling a deeper understanding of their experiences and possible implications for recovery.

## Method

### Systematic search

The current metasynthesis included three stages: 1) A systematic literature search of qualitative studies reporting women’s experiences of psychosis, 2) a critical appraisal of studies identified, and 3) the metasynthesis of these studies.

Published articles were identified through searches of the following databases: EMBASE (1980 to 2012), MEDLINE (1946 to 2012) and PsycINFO (1806 to 2012). The search terms in Table 1 were combined. The reference lists of all relevant articles were examined for additional studies that met the inclusion/exclusion criteria.

**Table 1:** **Final search criteria and terms**

**Women**	**Psychosis or bipolar disorder**	**Qualitative research**
Women	Serious mental illness	Qualitative
		Grounded theory
Mother	Severe mental illness	Phenomenology
		Narrative
Mothers	Psychosis	Discourse
		Ethnography
Mothering	Schizophrenia	Thematic
		Interpretative phenomenology interview
	Bipolar disorder	Focus group
	Bipolar affective disorder	Explorative
		Observational
		Constant comparative

### Inclusion and exclusion criteria

**Figure 1:**
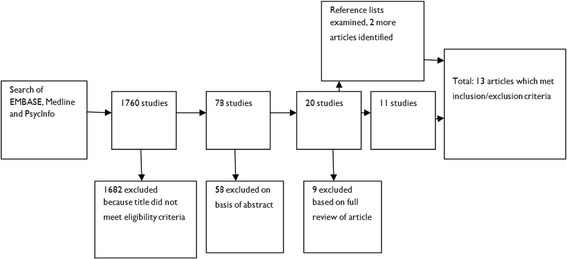
**Flow chart to illustrate results of search strategy.**

The combined search strategy yielded 1760 studies. All titles were screened and studies were included them if they were written in English and used qualitative methods and data analysis to explore the experiences of women with psychosis. During screening, diagnostic categories that were considered to be indicative of psychosis included the following: psychosis, psychotic experience/symptoms, depressive psychosis, puerperal psychosis, schizophrenia, schizophrenic disorder, schizoaffective disorder, bipolar disorder and bipolar affective disorder. Articles which studied women with various diagnoses were included if at least 50% of the sample comprised women with experience of psychosis. Studies were excluded if structured questionnaires were used as the only method of data collection or if only quantitative data were reported. Mixed method studies were included if the results from the qualitative methods were reported separately. Studies that did not report data from women themselves were excluded. The majority of studies were excluded after an initial screen of the title indicated that they were not relevant. If relevance remained ambiguous, the abstract was reviewed. All potentially relevant studies were reviewed in full to determine eligibility (see Figure 1).

### Critical appraisal

We used the Critical Appraisal Skills Programme (CASP) criteria [27], augmented by guidelines constructed by Walsh and Downe [28], to assess the quality of the identified studies. Each study was allocated a total score out of 10 and a corresponding classification. Studies in Category A received a score of at least 9 and were deemed to have a low risk of bias. Studies in Category B (scores between 6 and 8) were deemed to have a moderate risk. Studies (<6) were assigned to Category C and were deemed to have a high risk of bias.

An independent rater checked the studies using these criteria. The raters were in 89% agreement. When disagreement occurred, scores were reassigned following discussion. Scores for two of the 13 articles were adjusted, which did not affect the categories to which they were assigned.

All identified studies were included because even studies with less rigorous methodologies can be valuable in the synthesis process [29].

### Metasynthesis analysis

Noblit and Hare’s [29] method was used because it is one of the most developed and frequently used [30,31]. It allows for the preservation of the interpretative properties of primary data. Themes (i.e., third order constructs) are built from second order constructs, which are the views and interpretations of the authors expressed in terms of themes and concepts [31]. Tables, in which second order constructs from each article were illustrated by raw data in two columns, were explored for similarities, differences and relationships between the data. The following techniques were employed: 1) Reciprocal translation where concepts were translated into one another, 2) refutational translation, where contradictions between concepts were explored and finally, 3) synthesis of the translations to create overarching themes to explain the phenomena in the studies. In this iterative process, the phases described are not distinct but overlap and are repeated with progression of analysis [29-31].

## Results

### Quality assessment

Thirteen articles met the inclusion criteria (see Table 2). Five [32-36] of the 13 studies were based in the US, four [37-40] in Canada, three [41-43] in the UK, and one [44] in Japan. All of the studies used interviews to generate data, including semi-structured (n = 5) [33,34,36,38,39], in depth (n = 2) [35,40] and narrative (n = 2) [37,43]. In the remaining four studies [32,41-43], the type of interview was not specified. Two studies used observational methods to collect additional data [34,36]. Across the 13 studies, the experiences of 250 women were reported. Only nine studies reported the ages of participants (range: 21–73 years). Seven studies focused on women regardless of parental status, whereas six focused exclusively on mothers (n = 78, 31.2%). However, of the seven studies that focused on women, only two of them specified parental status.

**Table 2:** **Study characteristics of 13 studies**

	**Authors**	**Year**	**Country**	**Description of sample**	**N**	**Women/mothers**	**Ethnicity**	**Recruited from**	**Data collection**	**Analysis**	**Score & quality rating**
1	Hagen & Nixon [37]	2011	Canada	Women recovered from some form of psychotic experience. Diagnoses not stated. Age range: 27–57 years (mean: 38). No information about parental status. No information about level of education, religion, relationship status or SES.	18	Women	Not stated	Word of mouth/advertise-ment in alternative health magazine	Narrative interviews	Phenome-nological data analysis	7 B
2	Borba et al. [32]	2011	USA	Women with diagnosis of SMI (10 schizophrenia, 6 BD, 13 major depression and 1 other). Sample described as low income, urban women. Age range: 28–62 years (mean = 45.8). No information about parental status. Highest level of education: Less than a high school diploma (10), high school diploma (16), some college credits (4). Religion not stated. Marital status: Married or living as married (2), widowed (4), separated (3), divorced (7), single, never married (14). No information about religion.	30	Women	28 African American 1 White, 1 other.	Community settings	Interviews	Modified constant comparative method	8 A
3	Venkataraman & Ackerson [33]	2008	USA	Mothers with BD. Age range: 21–49 years (mean not stated). Education: High school (5), college (5). Relationship status: Single (6), married (1), cohabiting (3). SES: Low (7), middle (3). No information about religion.	10	Mothers	White American	Community settings	Semi-structured interviews	Constant comparative analysis	6.5 B
4	Luhrmann [34]	2008	USA	Homeless women with psychotic symptoms. No formal diagnostic interview. Ages not stated. No information about parental status. No information about education, religion, or relationship status.	61	Women	Not stated	Community settings	Semi-structured interviews/observation	Not stated	5.5 C
5	Ueno & Kamibeppu [44]	2008	Japan	Mothers with chronic mental illness (13 schizophrenia, 7 mood disorders). Age range not stated. (mean: 43). Relationship status: Married (14), separated or divorced (4), widow (1), never married (1). No information about education, religion or SES.	20	Mothers	Japanese	Discharged inpatient/community settings	Narrative interviews	Modified grounded theory	7.5 B
6	Chermonas, Clarke & Marchinko [38]	2008	Canada	Women with schizophrenia (9 schizophrenia, 3 schizoaffective disorder & 2 paranoid schizophrenia). Age range: 40–73 years (median: 45). No information about parental status. Education: Less than high school (4), completed high school (2), completed or some post-secondary education (8). Marital status: Married or living common law (3), single or divorced (9), have boyfriends (3). SES: Low (8), middle (4), high (2). No information about religion.	14	Women	Most White/White with European Ancestry, 1 part Aboriginal, 1 Asian.	Community settings	Semi-structured interviews	Not stated	6.5 B
7	Padgett et al. [35]	2006	USA	Formerly homeless women with SMI (5 schizophrenia, 2 schizoaffective disorder, 3 major depression, 3 BD). Age range: 31 – 62 years (mean: 50 years). 7 mothers. SES: Poor/working class (8), middle class (5). No information about education, religion or relationship status.	13	Women	6 African American 5 White, 2 Latina	Community settings	In depth life history interviews	Cross case/collective case study	9.5 A
8	Chiu et al. [39]	2005	Canada	Women with SMI (18 schizophrenia or schizophreniform disorder, 6 major depression, 4 BD, 2 major depression with psychosis/delusions). Age range: 26–67 years (mean: 46). No information about parental status. East Asian women: Education: Elementary school (1), high school (6), post-secondary education (7), no formal education (1). Religion: Christianity (7), Buddhism (2), Catholicism (1). Relationship status: Single (6), married (3), widowed (2), divorced (2). SES: Self-employed (2), on pension (2), welfare or disability (9), supported by families (2). South Asian women: Education: Elementary school (2), high school (5), post-secondary education (4), no formal education (4). Religion: Sikhism: 15. Sikhism with Hinduism: 2. Relationship status: Married (7), widowed (3), separated (2), divorced (3). SES: Employed (1), welfare or disability (5), supported by families (9).	30	Women	15 East Asian 15 South Asian	Community settings	Semi structured interviews	Constant comparative analysis	9.5 A
9	Edwards & Timmons [41]	2005	UK	Mothers with postnatal illness (3 puerperal psychosis, 2 severe postnatal depression, 1 depressive psychosis). Ages not stated. No information about education, religion, relationship or SES.	6	Mothers	Not stated	Discharged inpatient	Interviews	Not stated	7.5 B
10	Diaz-Caneja & Johnson [42]	2004	UK	Mothers with SMI (8 schizophrenia, 10 BD, 4 severe depression with psychotic symptoms). 2 mothers 20–29 years, 9 mothers 30–39, 11 mothers 40+. Marital status: Currently married/living with partner (3), previously married, now not living with partner (11), widow (1), never married (7). No information about education, religion or SES.	22	Mothers	13 White UK, 3 White other, 1 Black UK, 1 Black Caribbean, 1 Black African, 2 Asian, 1 Mixed	Community settings	Interviews	Thematic analysis	8.5 B
11	Robertson & Lyons [43]	2003	UK	Mothers with experience of puerperal psychosis. Age range 28–44 years (mean = 34). No information about education, religion, relationship status or SES.	10	Mothers	Not stated	Community settings	Interviews	Grounded theory	6 B
12	Pentland et al. [40]	2003	Canada	Aging women with schizophrenia (45+ years, diagnosis of schizophrenia in early adulthood). Age range: 47–65 years. 3 mothers. Marital status: Married (1), divorced (1), single (4). No information about education, religion or SES.	6	Women	Not stated	Community settings	In depth interviews.	Thematic analysis	6.5 B
13	Sands [36]	1995	USA	Mothers with SMI – single, low income mothers with chronic mental illness living in a supportive residential programme. (6 schizophrenia, 1 schizotypal personality disorder, 1 major depression, 1 BD, 1 unknown.) Age range: 22–40 years (mean = 27). Education: At least high school education (6), less than high school education (3), unknown (1). No information about religion.	10	Mothers	7 African American, 3 White	Community settings	Semi-structured interviews/observation	Not stated	5.5 C

Of the 250 women, 80 experienced psychosis, 77 schizophrenia and 34 bipolar disorder. Their diagnoses included the following: Psychosis (32%), puerperal psychosis (5.2%), schizophrenia (30.8%), bipolar disorder (13.6%), schizoaffective disorder (2%), major depression (10%) of which two women had severe postnatal depression, depression with psychotic symptoms (2.4%), schizotypal personality disorder (0.4%), and mood disorders (2.8%). In two cases (0.8%) diagnosis was not specified.

Three studies were assigned to Category A, eight to Category B, and two studies to Category C. Although all studies were included in the metasynthesis, the findings reported in articles assigned to Categories B and C were considered with more caution than those assigned to Category A. During the critical appraisal process, several weaknesses were commonly found. A detailed description of the sample and consideration of reflexivity were often lacking. Only eight of the 13 articles provided information about ethnicity and marital status of participants, seven included information about SES, five presented information about level of education and only one article provided information about religion.

### Synthesis

**Figure 2:**
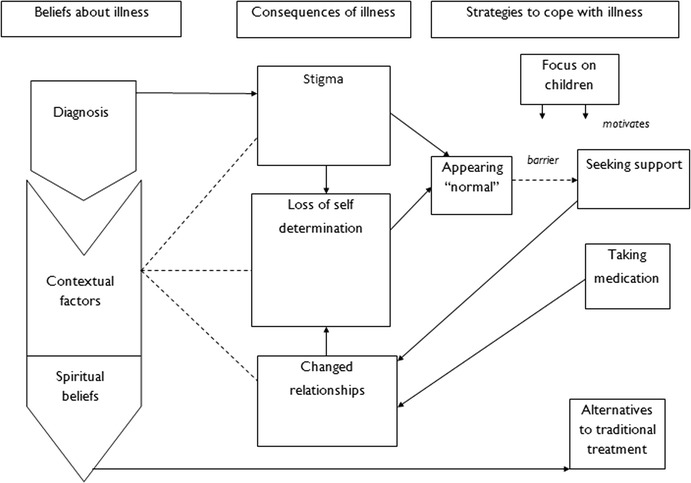
**A diagrammatic representation of the synthesis of articles.**

Three overarching themes were identified (see Figure 2): 1) women’s beliefs about their illness, 2) perceived consequences of illness, and 3) strategies used to cope with illness. Contextual factors and spiritual beliefs were found to be important themes in how women understood their experiences. Information related to diagnosis was incorporated into women’s models of illness only if it was concordant with their existing models. Women varied in the extent to which they accepted this information and found it useful. Women noted the negative consequences of their illness in relation to stigma, loss of self-determination and changes to relationships. These consequences interacted with one another and became ongoing contextual factors for women. They employed various strategies to cope with their illnesses. While the use of particular strategies augmented the use of others, in some cases strategies conflicted with one another. Strategy selection was driven by women’s beliefs about their illness and fear of consequences. Women discussed further changes in their relationships, which they attributed to their use of strategies (see Figure 2). Table 3 illustrates the third order categories noted in the identified 13 studies.

**Table 3:** **Studies illustrating third order constructs**

	**1**	**2**	**3**	**4**	**5**	**6**	**7**	**8**	**9**	**10**	**11**	**12**	**13**
	**Hagen & Nixon**	**Borba et al.**	**Venkataraman & Ackerson**	**Luhrmann**	**Ueno & Kampibeppu**	**Chermonas et al.**	**Padgett et al.**	**Chiu et al.**	**Edwards & Timmons**	**Diaz-Caneja & Johnson**	**Robertson & Lyons**	**Pentland et al.**	**Sands**
**Beliefs about illness**	Yes	Yes	Yes	Yes	Yes	Yes	Yes	Yes	Yes	Yes	Yes	Yes	Yes
Contextual factors	Yes	Yes	Yes	Yes	Yes	Yes	Yes	Yes	Yes	Yes	Yes	Yes	Yes
Spiritual beliefs	Yes	No	No	No	No	No	No	Yes	No	No	No	Yes	No
The role of diagnosis	Yes	No	No	Yes	No	No	No	No	Yes	No	No	No	No
**Consequences of illness**	Yes	Yes	Yes	Yes	Yes	Yes	Yes	Yes	Yes	Yes	Yes	Yes	Yes
Stigma: Feeling misunderstood/judged	No	No	No	No	No	Yes	Yes	Yes	Yes	Yes	Yes	No	No
Loss of self-determination	Yes	Yes	No	Yes	No	No	Yes	No	Yes	Yes	Yes	Yes	Yes
Changes to roles/relationships	No	Yes	Yes	No	Yes	Yes	Yes	Yes	Yes	Yes	Yes	Yes	Yes
**Strategies to cope with illness**	Yes	Yes	Yes	Yes	Yes	Yes	Yes	Yes	Yes	Yes	Yes	Yes	Yes
Focus on children	No	No	Yes	No	Yes	No	No	No	No	Yes	No	No	Yes
Seeking social support	Yes	Yes	No	No	Yes	Yes	Yes	Yes	Yes	Yes	Yes	Yes	No
Seeking support from professionals	Yes	Yes	Yes	Yes	No	Yes	Yes	Yes	Yes	Yes	Yes	Yes	No
Appearing “normal”	Yes	Yes	No	Yes	No	No	Yes	No	Yes	No	Yes	No	No
Taking medication	No	No	No	No	No	No	No	Yes	No	Yes	No	No	Yes
Alternatives to conventional treatment	Yes	No	No	No	No	No	Yes	Yes	No	No	No	Yes	No

### Theme 1: beliefs about illness

#### Contextual factors

Women believed that it was not possible to understand their experience of mental illness without reference to contextual factors [37]. The opportunities and difficulties, which women experienced, were strongly related to the social context in which they found themselves. Women’s social contexts included culture, religion and SES. A complex interaction between these factors and experiences including abuse and substance use, disruptive life events including loss of residence, and the need to fulfil role expectations, were noted [35]. For example, white and middle class women were more likely to have substance abuse problems than their African American and working class counterparts [35]. In addition, women who had somewhere to live that they perceived to be safe, had a stronger sense of personal agency [35].

Our synthesis also highlighted that the experience of immigration was important. Striving to fulfil role expectations was particularly pertinent for immigrant women who were required to adjust to new countries with different values and role expectations: “I got mentally sick because I was made to do a lot of household chores here. I have to look after four children plus do household chores. There was nobody to help me…” ( p. 638) [39].

Women came to understand that they had a responsibility to care for themselves [42] to remain able to care for their families [44]. Indeed women who were in a position to relinquish responsibilities associated with raising a family and engaging in paid employment discussed feelings of liberation [37]. Greater choice in relation to these responsibilities was mediated by factors, such as SES, culture and level of social support. For example, cultural differences existed in the nature of expectations for women to take up paid employment and share responsibility for raising children [39]. Further, the financial implications of loss of income were larger for women from lower SES groups, with lower levels of social support [32]. In addition to the role of contextual factors, some women held a stress-vulnerability model [45] of mental illness in which contextual factors caused illness in those with personal characteristics that rendered them vulnerable: “Some people cannot handle the pressure…They break and become mentally ill” ( p. 16) [34].

Women spoke of social isolation as an important factor contributing to and exacerbating their difficulties. Women discussed that professionals often ignored the impact of contextual factors in understanding their illness and their lives [37], which created a barrier to women seeking support from them: “If you don’t say, they don’t ask” ( p. 477) [42].

In contrast to women in other studies, the participants in one study [43] saw “the cause of their illness as biological” (p. 418). This finding might be explained by the fact that women were experiencing psychosis in the context of childbirth for which there is more evidence of a biological aetiology. However, because of methodological concerns associated with this study, this finding was given less weight.

#### Spiritual beliefs

Spiritual beliefs played a role in women’s models of mental illness [37], particularly the case for immigrant women. Ideas about the meaning of mental illness “varied with each person and from one culture to another” (p. 645) [39]. Women expressed representations of their experiences, which were directly related to their religious convictions, such as “a bad spirit resided in me” (p. 648) [39] or “a moving of energy” (p. 58) [37]. This impacted on women’s interpretations of their symptoms mediating the distress caused. For these women, it was not possible to recover until they had given due consideration to these factors [37], and often coping strategies were borne out of this understanding. Women found that a lack of interest and understanding from professionals regarding their spiritual beliefs was a barrier to seeking support [37].

#### The role of diagnosis

Women varied in their willingness to accept a diagnosis as part of their understanding of mental illness, which appeared to depend on whether or not a diagnosis was concordant with their existing model of mental illness. Women felt reassured and relieved that their symptoms were recognised as an illness, which could be treated [41]: a diagnosis could facilitate access to appropriate support and useful medication. However, some women actively avoided assessment and diagnosis despite potential benefits. For some, a lack of consideration of contextual factors or spiritual beliefs by health professionals rendered labels “meaningless” (p. 52) [37]. Luhrmann [34] discussed the importance of attempting to understand the meaning of a diagnosis within the context of the woman’s particular social world. Even for women who assimilated a diagnosis into their understanding of mental illness, they acknowledged stigma associated with this label [41].

### Theme 2: consequences of illness

#### Stigma: feeling misunderstood/judged

In relation to stigma associated with mental illness, women discussed the experience of perceived stigma from others [41,42] and self-stigma [39], including the restrictions of roles available to them resulting from stereotyping. This restriction contributed to their experience of social isolation [38]. However, women acknowledged that stigma associated with mental illness varied with culture: “The biggest comfort is that I am in Canada. It’s much better than in China. People like us in China would be stoned and stepped upon” (p. 641) [39].

Fear of stigma and the associated negative reactions of others motivated women to appear “normal”, which posed a barrier to seeking support both from informal support networks and professionals. Women’s fear of stigma was attenuated in relating to others who had experienced mental illness themselves [43].

#### Loss of self-determination

Women discussed a loss of self-determination during periods of illness, particularly in relation to hospitalization, when they perceived “having one’s freedom stripped away” (p. 55) [37], treatment [43] and threat of custody loss [41]. Loss of self-determination was associated with feeling “overwhelmed and powerless” (p. 56) [37]. Fear of loss of self-determination motivated women to appear “normal” [32] and prevented them seeking professional support.

Women also discussed a “lack of control” (p.289) over the contextual aspects of their life experience, such as abuse, financial disadvantage and drug use [32]. They spoke of the loss of being able to live independently [40] and dissatisfaction with the requirement to submit to restrictions imposed by providers of accommodation [34-36]. However, women expressed ambivalence as they also spoke about the benefits of “having their daily needs met without having to worry” [40].

#### Changes to roles/relationships

Women discussed changes to their relationships, which occurred as a result of their illness. Feelings of isolation led to power imbalances in relationships and associated vulnerability to “emotional and physical victimization” (p. 288) [32]. Women often felt that they had no choice but to remain in such relationships: “Sometimes we fight and he’ll get my schizophrenia involved…It makes me feel like I don’t know how to do anything…I feel defensive…I always say, ‘I’m not staying here anymore. I’m going to leave’, but I never do” (p. 445) [38].

After illness onset, women felt unable to fulfil certain role expectations [40]; trying to maintain certain roles contributed to stress and illness exacerbation [39]. Women described that relationships which had been happy previously, became strained [43] and sometimes broke down completely [32,40].

Mothers worried about the impact of their illness on their children [44]. Some acknowledged an “unhealthy dependency” (p. 394) [33] on their children during periods of illness and that children had taken on age-inappropriate responsibilities [42]. Mothers also recognised that symptoms [33,40,44] and medication [39,42] had an impact on their ability to parent effectively. These factors led to feelings of guilt, which motivated compensatory styles of parenting. Outcomes included inconsistent parenting practices, resulting in behavioural problems in children [33], which contributed to stress levels in mothers. Women who had lost custody of their children discussed the “emotional trauma” (p. 90) [36] associated with this loss [32,40].

### Theme 3: strategies to cope with illness

#### Focus on children

Women who were mothers described this role as rewarding and central to their lives [36]. They discussed that their children were a powerful incentive to recover and remain well [44]. This included motivating women to continue using other strategies that they perceived to facilitate this process; for example, continued engagement with professionals [42].

#### Seeking social support

Seeking social support was cited as an important factor in women’s ability to cope with mental illness [38,40]. Women valued having someone trustworthy to confide in who could provide practical and emotional support: “He’s a very supportive person; he loves me; and things that I feel I have to keep secret, I can talk to him about” (p. 445) [38]. Beliefs that their problems were exacerbated by social isolation motivated them to seek social support. However, barriers to accessing social support included an inability to be open about their experiences. Some were unable to work because of their illness so did not have this forum to meet people [38,40]. Women expressed the view that resources were inadequate to support them to continue with family and social life [39], leading to further family breakdown and social isolation. Women whose experiences had led them to believe they were “able to rely on few others for protection” were less likely to seek support (p. 17) [34]. Cultural differences affected the ways in which women used social support. For example, self-reliance in making treatment choices and the acceptability of separation or divorce from their spouse were factors that varied between cultures [39].

Women discussed the benefits of seeking support from others with similar experiences [30,43], believing that they would have more understanding of mental illness, be less likely to react negatively and able to offer normalising explanations. For some women this was the “most helpful part of their encounter with the mental health system” (p. 57) [37]. However, women also discussed difficulties in depending on others with mental illness, including the inconsistency of this support. Women recognised that “the support they wanted was not available when their friends were ill” (p. 445) [38] or frequently changing their place of residence [32].

#### Seeking support from professionals

Women discussed seeking support from professionals as an important aspect of coping with their mental illness [40]. This was particularly noted for women with impoverished social support networks: “He’s [psychiatrist] my biggest support…I can tell him my problems because he always listens” (p. 443) [38]*.*

Barriers to seeking support from professionals included a fear of loss of determination, stigma [39] and feeling “invalidated and unheard” by professionals (p. 53) [37]. Women discussed that a lack of continuity of care discouraged the formation of trusting relationships with professionals [38,42]. For women who had adapted to particularly hostile environments (i.e., homelessness), coping strategies were often in direct conflict with expectations of services [34]. Women who were parents discussed that this aspect of their lives was often ignored by professionals and that they needed more support in their parenting role [33]. An exception to this was in the study by Sands [36] who reported that although observations revealed that mothers “had difficulty attending to or disciplining” their children, they “did not acknowledge this need directly” and “acted as if they did not need help and had nothing to learn from the staff” (p. 93) [36]. A possible explanation for this was fear of custody loss associated with greater openness about their difficulties. However, less weight should be given to this finding because of the study’s low quality.

#### Appearing “normal”

Appearing “normal” to others was driven by a fear of losing self-determination, particularly in relation to hospitalization and custody loss. Appearing “normal” allowed women to avoid hospitalization because “when you started saying the right things, you would be considered better” (p. 55) [37]. This strategy was also driven by fear of stigma. For women who lived in more threatening environments (e.g., homelessness), appearing “normal” was a survival strategy because they believed that showing any sign of vulnerability would attract people who would take advantage of them [34,35]: “You have to keep your guard up at all times” (p. 17) [34]*.*

Women frequently moved house to hide their illness, contributing to social isolation [32]. Even women who viewed themselves as recovered from mental illness engaged in suppressing normal emotions driven by a fear that these would be viewed as pathological by others: “I was really conscious that you’re allowed…the blues…and I was thinking God even if I have that will they think I’m going downhill and put me on drugs” (p. 423) [43].

In addition to appearing “normal” to others, women also distanced themselves from others with mental illness [43]: “I can remember being in hospital…and I used to look at the other patients and think “blooming heck” what is up with them…and I was just as bad [she laughs]” (p. 476) [41]. For women whose illness was of a less chronic nature, the need to appear “normal” and to separate themselves from others with mental illness declined as they recovered. This was replaced by a desire to be open about their experience with the aim of reducing stigma [41]. It is possible that talking about their experiences enabled women to develop a narrative which facilitated their understanding of the experience and hence their recovery.

#### Taking medication

Women acknowledged the value of taking medication [36]. However, this was often an understanding that developed over time. Taking medication did not exclude the use of other strategies to cope. Women viewed strategies as serving different but complementary functions: “When I do meditation, I feel relaxed and my stress goes away. That’s the help I am getting from spirituality…medicine is also needed. Spirituality has its own values and medicine has got its own…both work hand in hand, not alone” (p. 648) [39].

#### Alternatives to conventional treatment

Women looked to sources other than the traditional mental health system to supplement their coping strategies [37]. Spirituality and belief systems were closely related to women’s choice of strategy selection [39]. Alternatives to traditional treatment included but were not limited to spirituality, religious practices, Reiki and yoga. For example, immigrant women engaged in culture-specific practices that often existed alongside engagement with conventional treatments [39]. Women discussed many benefits of spirituality including empowerment [35], acceptance, wisdom and strength: “I don’t think if I didn’t have God, I don’t think I could get through my life” (p. 296) [40]*.*

## Discussion

This is the first attempt to systematically explore the qualitative literature on the experiences of women with psychosis. Three overarching themes emerged and a model was developed to explain their experiences.

Women appear to construct idiosyncratic illness models of their experience, which did not necessarily incorporate information from dominant biomedical paradigms of mental illness. This is concordant with data suggesting that individuals’ understanding of their problems, rather than labels/diagnoses, have a strong mediating influence on how they view themselves in relation to mental illness [1,46].

Women were critical of health care professionals ignoring contextual factors in their lives. This is reminiscent of the feminist critique: labelling women as mentally ill hides injustices including social inequalities [12]. Gender interacts with other relevant variables, such as race, ethnicity and socioeconomic status. Women from minority groups (e.g., immigrants) are more likely than British born white women to experience mental ill health [12]. While racism, access to resources and social isolation might be important factors, there is evidence that their experience of the mental health system is different for these groups of women. For example, African-Caribbean women are more likely to be hospitalized on an involuntary basis and they are more likely to receive medication for mental distress [47]. The importance of the relationship between sociocultural factors with illness models has already been highlighted [46]: marginalized groups may be more likely to reject white, middle-class professionally conceived psychiatric explanations.

The way that mental health is conceptualised in western societies reflects underlying societal structures and relationships [12]. The mental health system reflects this bias and it has been argued that psychiatric care and treatment does not take account of spiritual belief systems and their role in coping with mental distress [48], as discussed by women in the current study. Religious beliefs might conflict with illness paradigms used by health professionals and could discourage engagement [49]. However, religion and spirituality might provide positive coping to people with bipolar disorder [49] and schizophrenia [50].

The role of self-determination in influencing health and wellbeing is well established. The failure to provide services, which preserve the self-determination of people with mental illness, has been reported elsewhere [51], but this problem is particularly pertinent for women [52]. Although a focus on self-determination is an important component of recovery-oriented services, it has been argued that these concepts have not yet been established in the mental health system [53]. Reducing women’s view that service involvement will inevitably lead to a loss of self-determination is likely to enhance engagement with services. Several recommendations have been made in the literature to help health professionals support the self-determination of people with mental illness, including a focus on individual preferences to inform decision-making [54] and paying attention to women’s beliefs about their illness rather than dismissing them in favour of dominant biomedical models [55].

Women reported that they believed an understanding of contextual factors to be imperative to understanding their mental illness and that professionals’ minimising of these factors discouraged engagement. The role of stress in precipitating psychosis has been established [45]; however, the link between contextual factors and psychosis might be particularly important for women because severe physical or sexual abuse was associated with psychosis in women but not men [13].

Although stigma is common to all mental illnesses, it is greater for people with SMI [56]. Stigma is not unique to women, but there is some evidence that the effect of stigma might be greater for women, having a greater impact on their willingness to disclose problems [57] and seek support [58]. Goffman [16] realised that stigmatized people reveal or hide certain parts of themselves in order to influence the reactions of others, demonstrated by women in the current study using the strategy of appearing “normal”. This strategy was driven by their beliefs about how others react to people with a mental illness in line with modified labelling theory [59]. Consistent with this approach, women who felt more threatened by the potential reactions of others were more motivated to hide their illness. Stigma and discrimination related to mental illness interact with additional inequalities related to gender and ethnicity to render some women doubly disadvantaged [60].

Changes in relationships were related to the restrictions imposed on social support networks caused by illness. However, the strategies people use to manage the reactions of others, such as social withdrawal and non-disclosure, can have negative consequences for social support networks [59]. Furthermore, individuals with psychosis may have a poor understanding of the feelings and intentions of others [61,62]. Thus, family members are not always equipped to provide the level of care needed by individuals with SMI [63].

As the process of metasynthesis is inherently interpretative, introducing subjectivity and potential bias, transparency in the development of themes was emphasised [64]. In addition, the systematic identification of studies and critical appraisal by two independent raters enhanced the rigour of the metasynthesis. Finally, comprehensive details of the included studies were provided to enable the reader to consider the findings within the various contexts. However, information about ethnicity, class, level of education, religion, marital and parenting status and SES were often not provided, but future research should routinely provide comprehensive information about male and female participants, including their parenting status. Notably, the majority of the participants in the 13 reviewed studies were described as poor.

As this metasynthesis focused solely on women’s views, it would be important for future research to synthesise the views of men so that similarities and differences between experiences could be evaluated. Some of the themes identified in this review may apply to men.

The findings outlined here have several implications for ways in which services can optimise women’s use of strategies for coping with mental illness. Given the importance of informal social support networks and the difficult changes in relationships that can occur as a result of illness, a family-centred approach should be taken to women’s care, by educating and supporting family members and involving them in decision-making. Professionals should be provided with regular training around how to enhance women’s engagement with services. Strategies might include ensuring continuity of care when possible, a more holisitic assessment of women’s difficulties which encompasses contextual factors and spiritual beliefs and inclusion of women in decision-making. Reflection on the assumptions of healthcare professionals about male and female behaviours and how this influences practice should be encouraged in supervision. If women believe they are listened to by culturally sensitive services, women will be able to seek more support, optimising their coping.

As some women are also mothers and parents, their wellbeing, their child(ren)’s wellbeing and that of the family should be enquired about, if possible assessed and, where necessary, appropriate and effective parenting interventions should be offered [65,66].

## Conclusion

In summary, women with psychosis hold beliefs about their illness, which incorporate contextual factors and spiritual beliefs in addition to biomedical explanations. They developed adaptive strategies to cope with their illnesses, which can be understood in the context of their beliefs.
